# A Summary of Ferrier’s Localizations

**Published:** 1879-07

**Authors:** 


### A SUMMARY OF FERRIER'S LOCALIZATIONS.

The following useful summary of localizations described by Dr. Ferrier in his Goulstonian Lectures on the Localization of Cerebral Diseases, (Smith, Elder & Co., 1878), is given in the Birmingham *Medical Review,* April 1879. It will probably be found useful and acceptable by a large number of readers.

*Lesions of Frontal Lobes.*—The frontal lobe includes the superior middle and inferior frontal convolutions, the ascending frontal convolutions, and the orbital and internal aspect of the same region, but for pathological purposes it is necessary to sub-divide this, and to describe that part which, in its relation to the skull, is roughly bounded by the coronal suture, as the prefontal lobe, or anterofrontal region. In the monkey, electrical stimulation of this region causes no motor reaction, and destruction of this region is followed by no paralysis of motion or sensation. There are numerous cases on record all pointing to the same conclusion ; most extensive injuries of these lobes having produced no paralysis of motion or sensation. There is reason to believe that their function is in some way bound up with the higher manifestations of intelligence, their deficiency being frequently associated with idiocy, and their removal in monkeys, leading to impairment of the faculty of attention and intelligent observation.

*Lesions on the Motor Regions.*—The motor area, as determined by experiments on monkeys, includes the bases of the three frontal convolutions, and those convolutions bounding the fissure of Rolando, viz. : the ascending frontal, the ascending parietal convolutions, the postero-parietal lobule, and the internal aspect of the same called the paracentral lobule. General or extensive lesions of this area are followed by paralysis of voluntary motion without affection of sensation on the opposite side of the body. This paralysis is frequently associated with rigidity or convulsive spasms in the paralyzed parts, particularly in the early stage ; and if destruction of the cortical substance be complete, the paralysis is of permanent duration, and sooner or later is followed by late rigidity and secondary sclerosis of the motor tracts, traceable down the crus and pyramid, and thence mainly in the opposite side of the cord in the posterior part of the lateral column, a corresponding band of secondary degeneration frequently existing in the internal aspect of the anterior column on the same side of the lesion. (In an earlier part of his book, p. 11, Dr. Ferrier refers to this incomplete decussation of the motor tracts as explaining the occasional departure from the rule that in cerebral hemiplegia the paralysis is on the opposite side of the lesion. He points out that according to Flechsig there is no normal fixed percentage of crossing and direct fibres, but that these present individual variations from three principal types, (*a*) total decussation (*b*) semi-decussation of one pyramid, (*c*) semi-decussation of both pyramids, while in rare cases the decussation of the pyramids may entirely fail). But what is true of lesions of the cortical substance holds good equally of the subjacent white matter, which consists, at least in part, of the direct motor fibres passing down from the grey matter.

Partial lesion of the motor area gives rise to hemiplegia, but as the area of anatomically demonstrated lesion is not necessarily coextensive with the area of functional disturbance, conclusions as to exact localization from a purely clinical point of view are always more or less doubtful. Thus, in one case the lower two-thirds of the ascending parietal convolution, in another the left paracentral lobule, were the seats of the lesion.

*Oculo-motor paralysis*, sometimes occurs independently. Dr. Ferrier has found that in the monkey there is an area at the base of the first frontal, and extending partly into the second frontal convolutions, irritation of which causes elevation of the eyelids, dilatation of the pupils, conjugate deviation of the eyes and turning of the head to the opposite side. There are some cases which indicate the existence of a similar centre in the corresponding part of the human brain, but the facts at present known are not decisive.

*Paralysis of the leg* may exist apart from that of the arm. In the mokey, stimulation of the postero-parietal lobule causes movements of the leg as in walking. There are cases on record in which the lesion has been found in this region, in the upper part of the ascending parietal convolution and in the paracental lobule. Dr. Ferrier says it is necessary to be cautious in drawing conclusion as to the exact position of the arm and leg centres in man from considerations of mere anatomical homology, but the cases referred to show a rough agreement between the data of the experiment and the facts of morbid anatomy. Where paralysis of the arm is associated with that of the leg the ascending frontal convolution is also implicated.

*Paralysis of the arm* only has been seen in a case where hæmorrhagic extravasation three millimetres in extent was situated at the upper part of the ascending frontal convolution. But the centre for the hand is placed by experiment in the ascending parietal convolution, which is remarkably confirmed by the examination of the brain, in a case, reported by Dr. Gowers, of congenital absence of the hand.

Paralysis of the arm is frequently associated with paralysis of the face. The lesions causing this affection are all towards the middle or lower third of the ascending convolutions, where experiments in monkeys have localized the facial and manual centres.

*Facial Paralysis* from cortical lesions unassociated with brachial paralysis or aphasia is uncommon, and is always on the right side. It is probably due to a lesion of the lower frontal convultions near their junction with the ascending frontal.

*Aphasia* is the result of destruction of the orolingual centres which are situated at the lower extremity of the ascending frontal, where it joins the third frontal, as is well known. Aphasia, in the great majority of instances, occurs only when the lesion is on the left side, and it is remarkable that in several at least of the cases in which aphasia has occurred with disease of the right speech centre, the patients have been left-handed.

In reference to the diagnosis of cortical paralysis, Dr. Ferrier remarks that apart from considerations as to the diathetic indications, mode of onset, etc., of the affection there are no features which clearly enable us to distinguish between hemiplegia depending upon general destruction of the motor area of cortex and hemiplegia due to the destructine lesion of the corpus striatum, more especially those involving the anterior two-thirds of the interior capsule. But hemiplegia, complete from the first and permanent, is not the most common type of paralysis depending upon cortical lesion; on the contrary, the affection is often limited or transitory, a hemiplegia passing into a brachial or crural monoplegia, or monoplegia with spasm. Early rigidity is of frequent occurrence, and consciousness is less frequently lost. As Callendar has pointed out, cortical lesions are more frequently accompanied by localized pain in the head. Irritative lesions of the motor area cause, not paralysis, but spasm, and the seat of the lesion may be approximately determined by the rules as to the localization of destructive lesions, but with more uncertainty, from the obvious difficulty of determining the area of the zone of irritation or the special point in this zone in which the irritation concentrates itself.

Destructive lesions of the posterior third of the internal capsule cause hemi-anæthesia of the opposite side of the body, a fact now well established. This hemi-anæthesia is accompanied be a defect or abolition of taste, smell, and hearing, with loss of visual acuity, contraction of the field of vision and color-blindness. If, as was formerly supposed, only the internal fibres of the optic tract decussate, there should be hemiopia of both eyes, and not amblyopia, as is the case. This difficulty is got over by assuming that the fibres which do not decussate in the chiasma do so in the corpora quadrigemina. "It is clear," says Dr. Ferrier, "that the lesion of the internal capsule which produces these effects does so by causing a solution of continuity of the paths of centripetal impressions," and he proceeds to enquire where those centres are to which these impressions are ultimately conveyed. Both experiment and morbid anatomy exclude the occipital lobes. Lesion of these are latent, though the author believes that these lobes are connected with our visceral impressions, animals who have suffered mutilation of these parts refusing to eat. We turn, therefore, to the temporo-parietal region, consisting of the supra-marginal lobule and angular gyrus in the parietal lobe, and all the convolutions of the temporo-sphenoidal to be on its external and internal surfaces. Lesions of this part in the lower animal produce impairment or paralysis of sensation on the opposite side of the body.

*Vision.*—Unilateral destruction of the angular gyrus in the lower animal is followed by temporary blindness in the opposite eye; bilateral destruction by permanent blindness in both eyes.

*Hearing.*—Destruction of the superior temporo-sphenoidal convolution on the side causes impairment of hearing on the opposite side; bilateral destruction causes complete deafness.

*Smell.*—Destruction of the lower extremity of the temporo-sphenoidal lobes on one side causes loss of smell on the same side, and if invading neighboring regions, causes loss of taste as well; bilaterial destruction causes complete loss of taste and smell.

*Common and Muscular Sensation.*—When the region of the hippocampus major and uncinate gyrus was ploughed up in such a manner as to avoid the internal capsule and medullary fibres of the other cortical regions (with the exception of the occipital lobe) tactile sensation was abolished on the opposite side: sight and hearing remaining unimpaired.

These are the results of experiments on animals. At present they are not fully confirmed by pathology, lesions in these regions being usually described as latent, but Dr. Ferrier says there is reason to believe that the latency may have been in the observation. In reference to cortical lesions and their effects on hearing, he quotes a case of what Kussmaul has called "word deafness," associated with softening of the first and second temporo-sphenoidal convolutions. In this condition of word deafness the patient may be able to hear but not understand spoken words, though he can read and write. He also quotes a case of word-blindness, an analogous affection of the visual centre, in which the lesion corresponded with the angular gyrus.

With regard to the localization of tactile sensibility, Mr. Jonathan Hutchinson concludes, from his observations on cranial injuries, that contusion of the sphenoidal lobe more particularly causes along with partial motor paralysis, paralysis of tactile sensation on the opposite side of the body.—*London Medical Record.*

